# Implementation of a Positive Technology Application in Patients With Eating Disorders: A Pilot Randomized Control Trial

**DOI:** 10.3389/fpsyg.2018.00934

**Published:** 2018-06-11

**Authors:** Angel Enrique, Juana Bretón-López, Guadalupe Molinari, Pablo Roca, Ginés Llorca, Verónica Guillén, Fernando Fernández-Aranda, Rosa M. Baños, Cristina Botella

**Affiliations:** ^1^Department of Basic, Clinical Psychology and Psychobiology, Universitat Jaume I, Castellón de la Plana, Spain; ^2^CIBER de Fisiopatología de la Obesidad y Nutrición (CIBEROBN), Madrid, Spain; ^3^Department of Personality, Assessment and Psychological Treatment, Complutense University of Madrid, Madrid, Spain; ^4^Psychiatry Service, Consorcio Hospitalario Provincial de Castellón, Castellón de la Plana, Spain; ^5^Department of Personality, Evaluation and Psychological Treatment, University of Valencia, Valencia, Spain; ^6^Department of Psychiatry, University Hospital of IDIBELL – Bellvitge Biomedical Research Institute, Barcelona, Spain

**Keywords:** eating disorders, positive psychological intervention, best possible self, optimistic thinking, affect, positive technology

## Abstract

**Background:** Positive psychological interventions (PPIs) have been suggested to produce benefits in patients with eating disorders (ED) by improving well-being, which might act as a buffer of the harmful effects caused by the disorder. Best Possible Self (BPS) is a PPI which consists of writing and envisioning a future where everything has turned out in the best possible way. In this regard, positive technology (PT) can be of considerable benefit as it allows to implement specific PPIs that have already shown efficacy.

**Objective:** This study tested the preliminary efficacy of the BPS exercise implemented through a PT application and carried out for 1 month, in improving positive functioning measures, compared to a control condition, in patients with ED. Follow-up effects were also explored at 1 and 3 months later.

**Methods:** This is a pilot randomized controlled trial, with two experimental conditions. Participants were 54 outpatients, who were receiving ongoing specialized treatment in ED services. 29 participants were randomly allocated to the BPS intervention and 25 to the control exercise. The sample was composed mostly by females and the mean age was 27 years. In the intervention group, participants had to write about their BPS. In the control group participants had to write about their daily activities. The exercise was conducted through the Book of Life, which is a PT application that allows users to add multimedia materials to the written content. Measures of future expectations, affect, dispositional optimism, hope and self-efficacy were assessed at different time frames.

**Results:** Findings showed that all participants improved over time and there were no statistically significant differences between conditions on the specific measures. These effects were not influenced by prior levels of ED severity. Within-group effect sizes indicate a greater benefit for the participants in the BPS condition, compared to the control condition, on nearly all the measures.

**Conclusion:** Results indicated that PT produced modest improvements in patients with EDs that are receiving current treatment for ED. More empirical attention is needed to explore the potential benefits of PPIs as supporting tools in the prevention and treatment of EDs.

**Trial registration:** clinicaltrails.gov Identifier: NCT03003910, retrospectively registered December 27, 2016.

## Introduction

Eating disorders are considered serious psychiatric disorders which cause functional impairment, emotional distress and different health problems, producing a negative impact in the quality of life of the patients (Hudson et al., 2007; Mond et al., 2012). It has been found that individuals with ED symptoms present higher levels of neuroticism and lower levels of life satisfaction and optimism compared to healthy peers (Brannan and Petrie, 2011; Góngora, 2014; Garcia et al., 2017). Various studies have shown that patients with ED have an impoverished self-concept characterized by many negative self-schemas and few positive ones, contributing to the formation and persistence of the disorder (Cash and Deagle, 1997; Fairburn et al., 2003; Claes et al., 2009). Consequently, these patients often have a pessimistic view of recovery, and they find it quite difficult to imagine a better future (Stein and Corte, 2007; Malson et al., 2011).

Regarding treatment, EDs are very difficult conditions to be treated and in many cases patients remain ill over years, becoming chronic patients (Geller et al., 2001; Noordenbos et al., 2002; National Institute for Clinical Excellence, 2017). In regards to evidence-based treatments for these conditions, only bulimia nervosa has been shown to be effectively treated with cognitive-behavioral therapy (CBT) showing strong effects, but the current evidence does not suggest any preference for any treatment in anorexia nervosa or non-specified EDs in relation to efficacy (Fairburn and Harrison, 2003; Fairburn, 2005). Given the limited efficacy of conventional treatments, a new treatment approach, called the recovery approach, has emerged with a change on the focus of treatment goals, from the full recovery and weight restoration, to the reestablishment of quality of life and well-being (Slade, 2010; Dawson et al., 2014). Thus, within this approach, patients are encouraged to be proactive, optimistic and decisions about treatment are taken collaboratively between patients and their practitioners (Turton et al., 2011). Based on this approach, one study (Touyz et al., 2013) adapted two existing psychotherapy protocols for severe patients with anorexia nervosa (CBT vs. specialist supportive clinical management) and compared the effectiveness by making quality of life the focus of the treatment, instead of weight restoration. Results showed that, even with the shift in the treatment goals, changes in weight restoration and symptom reduction were achieved, along with high retention rates at the end of the treatment.

The focus on well-being as a treatment goal emphasizes the role of positive emotions, which might act as buffers against the deleterious effects of ED symptomatology (Brannan and Petrie, 2011). Following this vein, some authors have proposed the inclusion of positive psychology strategies for the prevention and treatment of patients with ED (Steck et al., 2004; Kirsten and du Plessis, 2013). It is claimed that the development of interventions focused on improving well-being and meaning in life on patients with ED could act as a protective factor against the negative impact of ED symptoms and body dissatisfaction by promoting a more positive attitude toward the self (Brannan and Petrie, 2011; Góngora, 2014). Also, Tchanturia et al. (2015) suggest that the inclusion of PPI could play a role in recovery of patients with EDs by enriching current programs and even enhancing their impact. These PPIs have been found effective for depressive patients on improving subjective well-being and decreasing depressive symptoms (Bolier et al., 2013; Mongrain et al., 2015; Chaves et al., 2016). To our knowledge, there are no randomized control trials testing the effects of PPIs on well-being for ED patients. Indeed, only one pilot study showed that implementing a positive psychology group intervention in an ED impatient service with young females was feasible and participants benefited from the program (Harrison et al., 2015). However, the results, although encouraging, were preliminary in nature due to the lack of a control group and the small sample size consisted of eight young female inpatients.

Given the potential benefits of implementing PPIs in patients with ED, it is important to select those strategies with the greatest ability to produce benefits based on the needs of the individuals (Lyubomirsky and Layous, 2013). In the case of patients with ED, some authors suggest that therapeutic practices aimed to develop personally meaningful and optimistic views about recovery and reflect on a prospective self beyond the disorder might be of considerable benefit (Malson et al., 2011). In this line of research, PPIs have shown their effectiveness in improving optimism (Malouff and Schutte, 2016). Specifically, the review conducted by Malouff and Schutte (2016) brought to light that the most powerful exercise to enhance optimism levels was the BPS exercise (Malouff and Schutte, 2016). This exercise consists of thinking and imagining about a future in which everything has turned out as well as it possibly could (King, 2001; Sheldon and Lyubomirsky, 2006). Besides, this exercise has also been tested with depressive patients, showing that it is able to promote positive affect and life satisfaction, and to reduce depressive symptoms (Pietrowsky and Mikutta, 2012; Sergeant and Mongrain, 2015). Recent controlled studies conducted by our group explored the effects of this intervention in university students finding positive effects in terms of optimistic thinking compared to controls (Enrique et al., 2017a). Another controlled study with a similar design examined the effects of BPS in a sample of patients with fibromyalgia finding benefits on affect and optimism after 1-month training (Molinari et al., 2017). Given the promising findings observed in other populations and the importance of developing optimistic views about the future in patients with ED, BPS exercise can be of considerable benefit for patients with this disorder.

A recent movement within the positive psychology field is the combination of these evidence-based strategies with Information and Communication Technologies (ICTs). This movement is called PT and is presented as a scientific and applied approach that uses technology for improving the quality of our personal experience with the goal of enhancing well-being and resilience (Botella et al., 2012). It is argued that PT can influence the personal experiences at three different levels. First, PT has the ability to improve emotional quality through the generation of positive and pleasant experiences. Second, PT can produce engaging and self-actualizing experiences. Lastly, PT can also be used to improve social integration and connectedness (Gaggioli et al., 2017; Guillén et al., 2017). In fact, all previous studies testing the efficacy of BPS that were conducted by our group implemented this exercise through PT, and found high levels of acceptability by the patients (Enrique et al., 2017a; Molinari et al., 2017).

### Rationale of the Study

The present study outlines a first approximation of PT to the ED field by studying the preliminary efficacy of the BPS exercise on a sample of patients who are receiving ongoing treatment. The design and procedure followed in the present study is very similar to prior controlled studies conducted by our group, which also implemented a PT application (Enrique et al., 2017a; Molinari et al., 2017). We only focused on examining the effects of this intervention on building positive aspects as opposed to reduce the negative, since this is aligned with the use of PPIs in clinical populations (Meyer et al., 2012; Schueller et al., 2014). Therefore, the goal of this study is to test the efficacy of the BPS exercise implemented through a PT application on different positive functioning measures on a sample of patients with ED. To our knowledge, this is the first controlled study to test this intervention in a sample of patients suffering this pathology.

The first hypothesis is that patients will present higher scores of positive expectations and positive affect and lower scores of negative expectations and negative affect after a single session, compared to a control condition in a sample of patients with ED. The second hypothesis is that the observed changes after one session will remain after 1 month training, compared to a control group. Furthermore, it is expected that the exercise will have an impact in self-efficacy, dispositional optimism and hope. Lastly, because there is a lack of empirical evidence about the maintenance of the effects over time, we preliminarily explored the effects after 1 and 3 months follow-up.

## Materials and Methods

### Design

This is an experimental, repeated-measures pilot study with two independent groups. Participants (*N* = 54) were randomly assigned to two conditions: 29 participants who performed the BPS exercise and the other 25 performed the daily activity exercise (control condition). The random assignment of the participants to the BPS and the control condition was carried out by an independent researcher who had no knowledge about the study. Random allocation was performed through a randomization list created by the Random Allocation Software, version 1.0. To ensure the homogeneity of the two experimental conditions, randomization was stratified by the level of functional impairment (mild-moderate-severe) rated by the therapists (GAF). The participants did not know the characteristics of the different experimental groups.

The study was registered in the United States National Institute of Health Registration System^1^ with Clinical Trials Registration Number: NCT03003910. Moreover, the study was approved by the Research Ethics Committee of the Provincial Hospital of Castellón. The recruitment processes and the data collection took place from October 2014 to September 2015.

**FIGURE 1:**
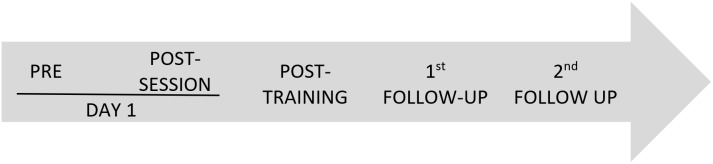
Assessment moments.

Assessments were conducted at five different moments (**Figure 1**): Prior to the exercise (pre), after the first session (post-session) and 1 month later (post-training). Moreover, two follow-ups were conducted 1 (1st follow-up) and 3 months (2nd follow-up) after finishing the training period (post-training).

### Participants

The total sample was composed by 54 participants, 52 women (96.3%) and 2 men (3.7%), who were recruited from four different outpatient psychology clinics where they were receiving treatment as usual for eating and personality disorders, mainly cognitive-behavioral therapy (Pike et al., 2015) and dialectical-behavior therapy (Linehan, 1993). Mean age was 27.1 years (*SD* = 8.6). Primary diagnoses of the patients were: ED (51.9%), or a comorbid diagnosis of eating and personality disorder (48.1%). Functional impairment of the patients was also collected, according to the GAF of the Diagnostic and Statistical Manual of Mental Disorders (4th Edition, American Psychiatric Association [APA], 2000) and they were categorized into mild (>60), moderate (51–60), and severe (<51). It is important to note that the diagnoses of the patients were based on the DSM-IV, given that they were collected from prior clinical records and the clinicians still used this classification. The GAF was measured by the personal therapist of each patient. 64.8% were categorized as mild, 31.5% as moderate, and 3.7% as severe in terms of functional impairment. **Table 1** includes detailed information of the participants regarding the assigned condition.

**Table 1:** Descriptive data about demographic variables, diagnosis, functional impairment and medication.

	BPS condition	Control condition	Total sample
**Age**			
Mean (*SD*)	27.65 (9.00)	26.44 (8.22)	27.1 (8.60)
**Sex**			
Male	1 (3.4%)	1 (4%)	2 (3.7%)
Female	28 (96.6%)	24 (96%)	52 (96.3%)
**Marital status**			
Single	24 (82.8%)	24 (96%)	48 (88.9%)
Married	3 (10.3%)	1 (4%)	4 (7.4%)
Divorced	2 (6.9%)	0 (0%)	2 (3.7%)
**Level of studies**			
Elementary school	1 (3.4%)	1 (4%)	2 (3.7%)
High school	10 (34.5%)	12 (48%)	22 (40.7%)
University degree	18 (62.1%)	12 (48%)	30 (55.6%)
**Diagnosis**			
Anorexia nervosa	4 (13.8%)	5 (20%)	9 (16.7%)
Bulimia nervosa	7 (24.1%)	4 (16%)	11 (20.4%)
Binge eating disorder	2 (6.9%)	2 (8%)	4 (7.4%)
EDNOS	16 (55.2%)	14 (56%)	30 (55.6%)
**Functional impairment**			
Mild	19 (65.5%)	16 (64%)	35 (64.8%)
Moderate	9 (31%)	8 (32%	17 (31.5%)
Severe	1 (3.4%)	1 (4%)	2 (3.7%)
**Medication**			
No medication	16 (55.2%)	12 (48%)	28 (51.9%)
Only anxiolytics	0 (0%)	1 (4%)	1 (1.9%)
Only antidepressants	2 (6.9%)	1 (4%)	3 (5.6%)
Only antiepileptics	1 (3.4%)	1 (4%)	2 (3.7%)
Only antipsychothics	0 (0%)	0 (0%)	0 (0%)
Combination of medications	10 (34.5%)	10 (40%)	20 (37%)


**BPS, Best Possible Self; EDNOS, Eating Disorder Not Otherwise Specified; Combination of medications: Any combination that include more than one type of medication*.*

#### Inclusion and Exclusion Criteria

The inclusion criteria used to select the participants were: (1) Aged between 18 and 70 years old, (2) Not suffering from a severe physical illness, (3) Not suffering from substance dependence, (4) Suffering from an ED condition.

### Measures

#### Primary Outcomes

##### Positive and negative expectations

We used the Spanish adaptation of the SPT (MacLeod, 1996; Dragomir-Davis, 2014). This instrument measures positive and negative expectations about events that will occur in the future. It consists of 30 items, 20 of them related to negative expectations about events that can take place in the future and 10 referring to positive expectations. The instrument asks individuals to judge the likelihood of an event happening in the future on a 7-point scale (from 1 “Not at all likely to occur” to 7 “extremely likely to occur”). Some studies have found appropriate internal consistency levels for positive and negative expectations (α = 0.80–0.82 and 0.91, respectively (Peters et al., 2010; Meevissen et al., 2011).

##### Positive and negative affect

To measure affect, we used the Spanish adaptation of the PANAS (Watson et al., 1988; Sandín et al., 1999). This instrument is composed of 20 items: 10 items measuring positive affective states and 10 items measuring negative affect states. Participants rate on a five-point scale (from “Not at all” to “Extremely”) the degree to which they usually feel a specific affective state. PANAS is one of the most widely used instruments to measure affect because it shows excellent psychometric properties (Cronbach Alpha’s from 0.87 to 0.91).

#### Secondary Measures

##### Dispositional optimism

We used the Spanish adaptation of the Life Orientation Test (LOT-R; Scheier et al., 1994; Otero-López et al., 1998). This scale measures the extent to which a person generally expects favorable outcomes. It includes 10 items: 3 items refer to positive expectations, 3 items refer to negative expectations, and 4 items are fillers. Answers are rated on a 5-point scale (from 0 “strongly disagree” to 4 “strongly agree”). Higher scores reflect a higher level of dispositional optimism. Other studies have found an internal consistency for the eight items of α = 0.76 (Meevissen et al., 2011).

##### Self-efficacy

The Spanish version of the GSES-12 was used (Bosscher and Smit, 1998; Herrero et al., 2014). This questionnaire evaluates general aspects of self-efficacy. The internal consistency coefficient for the scale is appropriate (α = 0.86).

##### Dispositional hope

It was used the Spanish version of the DHS (Espinoza et al., 2016). This instrument evaluates dispositional hope. It is composed of 12 items, with an 8-point Likert scale. It has shown good psychometric properties (α = 0.89).

#### Psychopathology Measure

##### Eating attitudes

We used the shortened Spanish version of the EAT-26 (Garner et al., 1982). This is a self-report measure that assesses disordered eating behaviors and attitudes. It is composed by 26 items rated following a 6-point Likert scale, in which “never,” “rarely,” and “sometimes” are scored as 0, “often” is 1, “usually” is 2 and “always” is 3. Higher scores indicate greater eating pathology. Scores of 20 or more indicate elevated risk of ED pathology. The instrument has shown excellent psychometric properties (Toro et al., 1989; Rivas et al., 2010).

### Positive Technology Applications

#### The Book of Life

**FIGURE 2:**
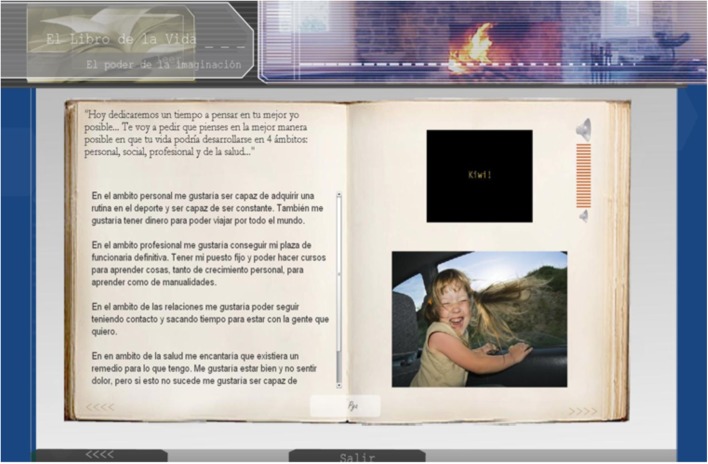
Screenshot of the positive technology application, Book of Life (embedded picture provided by Shutterstock, ID: 60967345).

It is a virtual application that seems like a personal diary and it is composed of different chapters where users are asked to write about different topics each targeting different psychological resources (Baños et al., 2014; Botella et al., 2016). Multimedia content such as audio, images and videos, can be added in order to enrich the experience and enhance the content of what they had written. For the purposes of this study, a new chapter was created with the instructions of the BPS exercise (Meevissen et al., 2011). Book of Life is a module of a self-applied technological system called EARTH, which, as a whole, has been proven effective in inducing positive moods (Botella et al., 2016). **Figure 2** illustrates a screenshot about how the exercise is displayed once the users have developed the content and selected the multimedia content.

#### TEO (Emotional-Online Therapy)

It is a web-based system that allows patients to do their homework assignments at home through the Internet (Quero et al., 2012). TEO permits clinicians to develop personalized therapeutic materials supported by multimedia content and share it with the patients in a simple and effective way^2^. In the present study, the exercise developed by the participants on the Book of Life was uploaded to TEO, including the multimedia materials, so that patients could practice it at home.

### Interventions

#### Best Possible Self

Participants in this condition were asked to write and imagine about a future in which all has gone in the best possible way and they have reached all their goals in four different domains: personal, professional, social and health domains. Participants were asked to develop the exercise through the Book of Life, where they could support the content they had written with multimedia content. Thereafter, this content was uploaded to the website TEO, so that they could access to this content with their own username and password.

#### Daily Activities (Control Condition)

Participants in this condition were asked to report activities, thoughts and feelings that had happened in the past 24 h. They were told that this exercise would help them to identify problematic areas in their lives and work on improving them. These instructions were adapted from other studies (Sheldon and Lyubomirsky, 2006; Meevissen et al., 2011). Participants in this condition were provided with a PowerPoint document where they wrote about the daily experiences, feelings and thoughts that happened to them in the last 24 h. The first slide included the instructions of the exercise and participant could add as much slides as they wanted.

All participants were given 20 min to complete the exercise. They were encouraged to write the content of the exercise in the format of a personal story to facilitate the visualization.

### Procedure

Sample recruitment was carried out by contacting the different coordinators of the outpatient clinical services. These clinics were specialized in the treatment of EDs and they were told that the intervention could have a positive influence on patients’ mood. Thereafter, the coordinators explained the information of the study to the psychologists at these units and they informed about the study to those patients who met the inclusion criteria. Thus, patients were explained about the features of the study and, if they agreed to participate, they were enrolled in a list of potential participants together with contact information. The experimental sessions were carried out in the clinical centers or in the university (depending on the preference of the patients) and they were carried out by the researchers. Patients were contacted by the researchers in order to make an appointment for the first session. When participants arrived, they were explained about the study and they signed an informed consent stating that they participated in the study voluntarily. Next, they were briefly screened about demographic information and completed the pre-test assessment (primary, secondary, and psychopathology measures). After that, the participants received the instructions for the corresponding exercise in audio format and on paper. For the performance of the exercise, participants on the BPS condition used the computerized program through a laptop provided by the researchers and the participants on the control condition used a PowerPoint file using the same computer. Then, participants were left alone in the room in order to avoid distracters and stimulate concentration on the exercise. All participants in both conditions prepared the exercise during 20 min. In the case of BPS condition, if multimedia content was not still selected, participants were encouraged to do it, allowing them to spend a maximum of 5 min. When the established time was over, participants of both conditions were asked to perform a 5-min visualization exercise in which they imagined their written BPS essay or their daily activities of the past 24 h. Specifically, participants of the BPS condition performed the imagery exercise through another display of the book of life, where they visualized the content of the exercise together with the multimedia content selected previously (**Figure 2**). In the case of the control condition, participants were also asked to read and visualize the content of their essays through the full screen mode of the PowerPoint file, in order to reproduce a similar methodology in both conditions.

To end the session, all the participants completed again the PANAS and SPT questionnaires with the items disorganized to reduce repetition effects. Furthermore, participants of both conditions were asked to practice the visualization exercise 5 min a day during a 1-month period. During this training period, two weekly text messages were sent to the participants’ mobile phones in order to remind them to perform the exercise. The content developed by the participants during the first session was either uploaded to the website TEO with the multimedia content in the case of patients in the BPS condition or sent by email in the case of patients in the control condition (powerpoint file). This was to allow participants continuous access to the exercises from their own homes.

At the end of the month, participants were given a second appointment to complete the post-assessment (primary and secondary outcomes). Finally, a follow-up assessment (primary and secondary outcomes) was conducted online 1 and 3 months after the post-training. During the follow-ups, all participants were encouraged to continue practicing the exercise at their own pace and they were told to practice at least 2 or 3 days per week to ensure that they would continue practicing. Besides, a text message was sent once a week until the end of the follow-up period.

### Data Analysis

Paired *t*-tests and chi-squared tests were conducted to explore the existence of significant differences in socio-demographic variables and baseline measures between conditions. CONSORT guidelines were followed to ensure the methodological quality of the study (Schulz et al., 2010). Missing data were treated following the procedure suggested by Hair et al. (2013). First, it was explored the type of missing data observing that it was at a construct-level and, thus, susceptible for imputation. Second, the quantity of missing values for each of the measures was explored, determining that none of the measures exceeded the recommended limits (Arias et al., 2015). Third, a diagnosis of the random pattern of missing data was carried out with the Little MCAR test (χ^2^ = 60.98; *p* > 0.05), concluding that missing data were completely at random. Lastly, intention to treat (ITT) analyses were carried out using Maximum Likelihood (ML) estimation performed via Expectation Maximization (EM) imputation method and sensitivity analyses comparing results of completers with the estimated values were conducted. These comparations showed that there was no chance of falling into biased estimations by using the ML estimation.

Before conducting the main analyses, correlations between ED severity, measured through the EAT-26, and the change in the outcome measures were conducted in order to explore whether severity was influencing the results. Thereafter, three sets of analyses were conducted to test the specific hypothesis. To test the first hypothesis, single-session effects (pre/post-session) were examined through analyses of covariance (ANCOVA; with condition as the between-subjects variable and pre-session scores as the covariate) to compare the effects of the intervention on affect and future expectations (primary outcomes) in the BPS and DA conditions. To test the second hypothesis, ANCOVA analyses (using condition as the between-subject factor and pre-session scores as the covariate) were carried out to explore the efficacy of the intervention at post-training for each primary and secondary outcomes. Finally, the effects of the intervention over time (pre, post-training, 1 month follow-up, 3-month follow-up) were examined by carrying out a 2 × 4 mixed ANOVA for each measure. All the assumptions for the ANOVAs performed were checked. In the case of mixed 2 × 4 ANOVAs, the degrees of freedom were corrected using Greenhouse–Geisser in those cases where the sphericity assumption was not fulfilled. Bonferroni correction was used for multiple comparisons. Effect sizes (Cohen’s *d* Cohen, 1992; Botella and Sánchez-Meca, 2015) and confidence intervals were calculated for within-group changes.

All statistical analyses were conducted using IBM SPSS Statistics 22.

## Results

### Participants Flow

**FIGURE 3:**
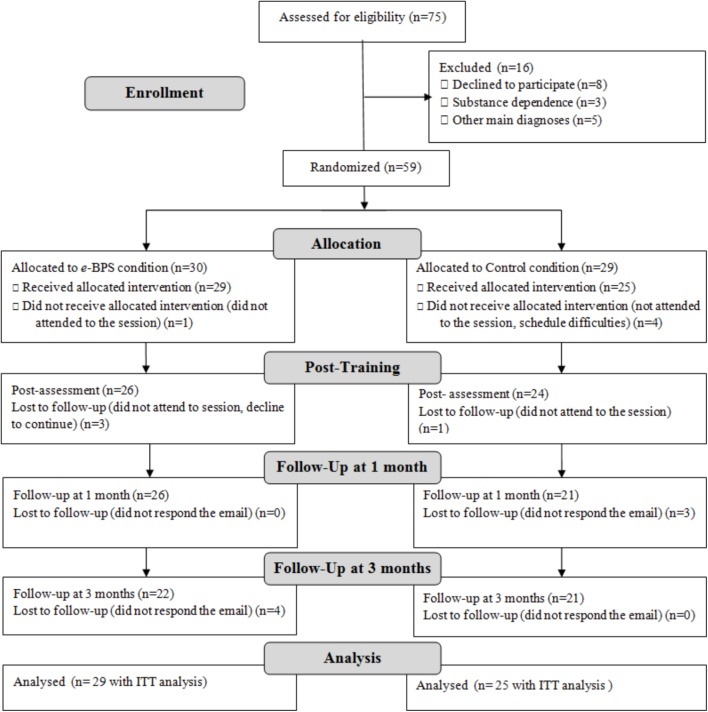
Participant flow (following consort flow diagram 2010).

Of the 75 patients initially included on the list of potential participants, 59 met the inclusion criteria, and they were randomly allocated to the conditions (**Figure 3**). Finally, the total sample receiving the allocated intervention was composed of 54 participants. During the training, the drop-out rate was 24.1% in the BPS condition and 16% in the control condition and these rates were slightly lower to those obtained in a prior study conducted by our group (26.3% in the BPS condition vs. 20% in the control condition; Enrique et al., 2017a). A total of seven participants did not respond to the online assessments at the follow-ups. There were no significant differences in drop-out rates between groups, χ^2^(1,54) = 0.55, *p* = 0.46.

### Pre-treatment Data

We first explored differences between groups at pre-treatment on any demographic variables, diagnosis, functional impairment and medication. The statistical analyses did not found significant differences between conditions on any of these variables. Correlations between EAT-26 and the change on primary and secondary measures at the different time points were conducted. None of these correlations were significant (*p* > 0.05), indicating that ED severity was not related to the changes in the outcome measures.

Regarding the frequency of practice, participants practiced on average 4.96 days per week over the training period (*SD*: 1.96) and this frequency decreased among the first (*M* = 2.94 days, *SD* = 2.03) and second follow-up (*M* = 2.40 days, *SD* = 1.73). There were no differences between conditions in the frequency of practice in the post-training and the first follow-up; however, in the second follow-up the participants on the BPS condition reported a significant higher frequency compared to controls [*t*(45) = 2.28, *p* = 0.03].

### Single-Session Effects

**FIGURE 4:**
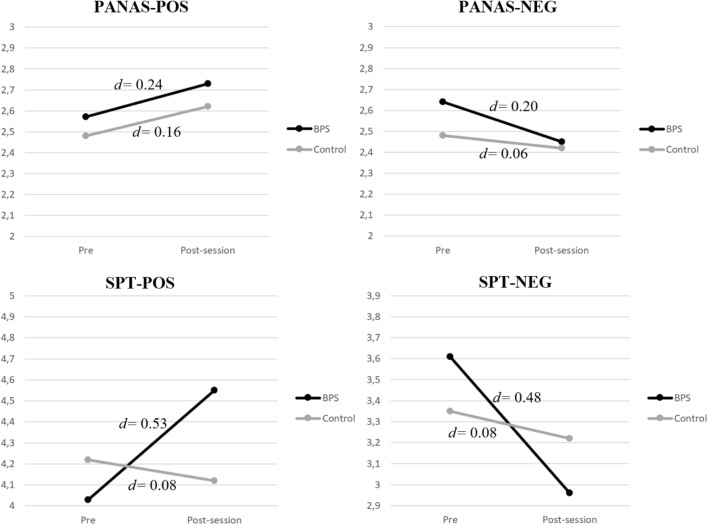
Single session effects on SPT and PANAS subscales separated by conditions.

ANCOVAs analyses on the baseline-corrected post-session scores showed a statistically significant effect of condition for positive [*F*_(1,51)_ = 9.88, *p* < 0.01] and negative expectations [*F*_(1,51)_ = 8.58, *p* < 0.01], revealing that there was a significant increase in positive expectations and a significant decrease in negative expectations in the intervention group compared to controls. In the case of affect, ANCOVA analyses did not show any significant condition effects on post-session changes for positive [*F*_(1,51)_ = 0.04, *p* > 0.05] and negative affect [*F*_(1,51)_ = 0.74, *p* > 0.05] subscales. **Figure 4** shows the graph of the change in scores for BPS and control conditions as well as the effect size for both measures. As the figure shows, both positive and negative future expectations revealed a significant moderate effect size (*d* = 0.53, 95% CI 0.25 to 0.81; *d* = 0.48, 95% CI 0.26 to 0.71, respectively) in the BPS condition, whereas no effect was found in the control condition. Regarding affect, participants in the BPS condition reached a significant small effect size (*d* = 0.24, 95% CI 0.02 to 0.45) for positive affect and a non-significant small effect size for negative affect (*d* = 0.20, 95% CI -0.02 to 0.39). In the control condition, both the positive and negative affect subscales revealed non-significant effects.

### Post-training Effects

The ANCOVAs on the baseline-corrected post-training scores were conducted for the different measures included in the post-training assessment, namely future expectations (positive and negative), affect (positive and negative), dispositional optimism, dispositional hope and general self-efficacy. Analyses showed a marginally significant condition effect for negative expectations [*F*_(1,51)_ = 3.74, *p* = 0.06], suggesting larger decreases in negative expectations in the BPS group, compared to the control group at post-training. Regarding the rest of the measures, ANCOVA analyses did not show any significant condition effect for positive expectations [*F*_(1,51)_ = 0.11, *p* > 0.05], positive affect [*F*_(1,51)_ = 0.91, *p* > 0.05], negative affect [*F*_(1,51)_ = 0.01, *p* > 0.05], dispositional optimism [*F*_(1,51)_ = 0.83, *p* > 0.05], dispositional hope [*F*_(1,51)_ = 1.06, *p* > 0.05] and general self-efficacy [*F*_(1,51)_ = 0.21, *p* > 0.05].

Focusing on within-group effect sizes, comparing pre-to post-training (**Table 2**), the improvements were more pronounced in the BPS condition. The average effect size for the different outcomes when comparing pre-post was 0.28 for the BPS condition and 0.18 for the control condition. Regarding the effect size in the specific measures, a moderate effect size was found for the BPS condition on negative future expectations (*d* = 0.57), while no effect was found for the control condition on this measure. Low effect sizes were observed on negative affect for both conditions, BPS (*d* = 0.34) and control (*d* = 0.37) groups. Besides, a low effect size was observed on positive affect for the control group, while this effect was not observed in the BPS group.

**Table 2:** Means, standard deviations and within-group effect sizes for the outcome measures in the different time-point assessments.

		Mean (*SD*)		Within-group effect size, *d* [95% CI]	Mean (*SD*)		Within-group effect size, *d* [95% CI]	Within-group effect size, *d* [95% CI]
		
		Pre	Post-training	Pre—post- training	1st follow-up	2nd follow-up	Pre—1st follow-up	Pre—2nd follow-up
SPT-POS	BPS	4.03 (0.95)	4.12 (1.44)	0.09 [-0.32-0.50]	4.67 (1.08)	4.81 (1.17)	**0.66 [0.23–1.08]**	**0.80 [0.31–1.28]**
	Control	4.22 (1.20)	4.37 (1.46)	0.12 [-0.20-0.44]	4.53 (1.42)	4.84 (1.10)	**0.25 [0.02–0.48]**	**0.50 [0.02–0.98]**
	Total	4.12 (1.06)	4.24 (1.45)		4.60 (1.24)	4.83 (1.13)		
SPT-NEG	BPS	3.61 (1.31)	2.84 (1.12)	**0.57 [0.21–0.92]**	3.32 (1.42)	3.07 (1.29)	0.22 [-0.11–0.55]	**0.40 [0.08–0.72]**
	Control	3.35 (1.51)	3.11 (1.60)	0.15 [-0.04–0.35]	3.30 (1.61)	3.28 (1.61)	0.03 [-0.18–0.24]	0.04 [-0.15–0.23]
	Total	3.49 (1.40)	2.97 (1.35)		3.31 (1.50)	3.17 (1.44)		
PA	BPS	2.57 (0.64)	2.69 (0.98)	0.18 [-0.12–0.49]	2.94 (0.91)	2.95 (0.96)	**0.56 [0.16–0.96]**	**0.58 [0.12–1.03]**
	Control	2.48 (0.86)	2.79 (0.96)	**0.35 [0.07–0.62]**	2.89 (1.15)	2.99 (0.88)	**0.46 [0.12–0.80]**	**0.57 [0.06–1.09]**
	Total	2.53 (0.74)	2.74 (0.96)		2.92 (1.02)	2.97 (0.92)		
NA	BPS	2.64 (1.00)	2.29 (0.84)	**0.34 [0.06–0.62]**	2.26 (0.98)	2.19 (0.81)	**0.37 [0.09–0.65]**	**0.44 [0.05–0.82]**
	Control	2.48 (0.84)	2.16 (0.91)	**0.37 [0.07–0.67]**	2.27 (1.07)	2.30 (1.07)	0.24 [-0.06–0.55]	0.21 [-0.08–0.50]
	Total	2.56 (0.93)	2.23 (0.87)		2.27 (1.01)	2.24 (0.93)		
LOT-R	BPS	18.24 (5.16)	18.94 (4.55)	0.13 [-0.24–0.5]	19.27 (5.10)	19.68 (5.27)	0.19 [-0.04–0.43]	0.27 [-0.01–0.55]
	Control	18.84 (5.31)	18.23 (6.76)	-0.11 [-0.39–017]	18.87 (6.84)	19.49 (6.01)	0.01 [-0.28–0.29]	0.12 [-0.25–0.49]
	Total	18.52 (5.19)	18.61 (5.64)		19.09 (5.92)	19.59 (5.57)		
DHS	BPS	40.58 (10.68)	44.17 (9.31)	0.33 [-0.02–0.67]	43.76 (9.76)	42.92 (11.78)	0.29 [-0.03–0.61]	0.21 [-0.09–0.52]
	Control	42.16 (13.32)	42.89 (12.64)	0.05 [-0.22–0.33]	43.40 (11.77)	43.84 (14.10)	0.09 [-0.21–0.39]	0.12 [-0.28–0.52]
	Total	41.31 (11.88)	43.58 (10.88)		43.59 (10.63)	43.35 (12.79)		
GSES	BPS	39.71 (6.66)	41.99 (6.94)	**0.33 [0.03–0.63]**	41.76 (7.54)	41.82 (7.54)	0.30 [-0.09–0.69]	0.31 [-0.06–0.67]
	Control	37.12 (9.19)	40.73 (10.16)	**0.35 [0.04–0.66]**	39.63 (10.89)	41.80 (7.81)	0.14 [-0.14–0.41]	**0.40 [0.02–0.78]**
	Total	38.51 (7.97)	41.40 (8.52)		40.77 (9.21)	41.81 (7.59)		


**Values marked in bold indicate significant effect sizes based on the Confidence Intervals (CI), which do not include zero. SPT-POS, Positive expectations; SPT-NEG, Negative Expectations; PA, Positive Affect; NA, Negative Affect; LOT-R, Life Orientation Test; DHS, Dispositional Hope Scale; GSES, General Self-Efficacy Scale; BPS, Best Possible Self.**

### Follow-Up Effects

To explore the effects of the intervention on the long-term, a 2 × 4 ANOVA analyses were conducted for each of the measures.

Regarding affect, analyses did not show interaction effects on positive [*F*_(2.41,125.35)_ = 0.24, *p* > 0.05] and negative affect [*F*_(3,156)_ = 0.74, *p* > 0.05] subscales. However, there was a significant time effect on both positive [*F*_(2.41,125.35)_ = 5.95, *p* < 0.01] and negative affect [*F*_(3,156)_ = 4.58, *p* < 0.01]. Regarding the latter, although the interaction effect was not statistically significant, pairwise comparisons revealed statistically significant differences between the pre- and first (*p* < 0.05) and second follow-up (*p* < 0.05) for the intervention group, while these effects were non-significant for the control condition. In the case of future expectations, ANOVA analyses did not show interaction effects between conditions for positive [*F*_(3,156)_ = 0.51, *p* > 0.05] and negative future expectations [*F*_(3,156)_ = 1.83, *p* > 0.05], but there was a time effect in both, positive [*F*_(3,156)_ = 6.99, *p* < 0.01] and negative future expectations [*F*_(3,156)_ = 5.58, *p* < 0.01]. In this regard, pairwise comparisons for positive future expectations, showed significant improvements from pre- to first follow-up (*p* < 0.05) and from pre- to second follow-up (*p* < 0.05) in the intervention group, while no significant effects were found in the control condition. Likewise, pairwise comparisons for negative future expectations showed a significant decrease from pre to second follow-up (*p* < 0.05) and from post to first follow-up (*p* < 0.05), and these effects were not found in the control group. Regarding other measures no interaction effects were found for dispositional optimism [*F*_(2.60,135.14)_ = 0.51, *p* > 0.05] and hope [*F*_(2.33,121.26)_ = 0.47, *p* > 0.05], neither time effects were observed. Lastly, results in general self-efficacy again did not show interaction between conditions [*F*_(3,156)_ = 0.76, *p* > 0.05], although a significant time effect was observed [*F*_(3,156)_ = 5.34, *p* < 0.01].

Focusing on the size of the change observed over time in terms of effect sizes, it is depicted in **Table 2**. On the 1st follow-up, the average effect size of the different outcomes was *d* = 0.37 for the BPS condition and *d* = 0.17 for the control condition. Likewise, at the 2nd follow-up, an average effect size of 0.43 was found for the BPS condition, and 0.28 for the control condition. Regarding the primary outcome measures, in the 1st follow-up, the positive subscales (SPT-POS and PANAS-POS) reached moderate effect sizes in the BPS condition (*d* = 0.66 and *d* = 0.56, respectively), and small to moderate in the control condition (*d* = 0.25 and *d* = 0.46, respectively). At the 2nd follow-up, in the case of SPT-POS, a large effect size was found for the BPS condition (*d* = 0.80), in contrast to a moderate effect size for the control condition (*d* = 0.50). Likewise, on the negative subscales, at the 2nd follow-up (SPT-NEG and NA), only the BPS condition reached a moderate effect size. Regarding secondary outcome measures, LOT-R and DHS showed a small effect size in the BPS condition in the different time frames, in contrast to the control condition, which did not produce observable effects. Finally, the effect size for GSES-12 was small for both conditions, with similar results.

## Discussion

This is the first pilot study randomized control trial to test the efficacy of a positive psychological exercise, the BPS, implemented through a PT application in a sample of patients with ED. The BPS exercise was tested again an active control group. Overall, the intervention produced a modest impact on the positive functioning outcomes included in the trial. The effects were more notorious at short-term, mainly in terms of future expectations, and these effects were vanishing over time.

Regarding the first hypothesis, it is partially confirmed. Results indicate that participants in the BPS condition significantly improved their levels of optimistic thinking compared to those in the control condition. However, these differences were not statistically significant for positive and negative affect, although in the case of positive affect, participants in the BPS condition reached a significant small effect size. These results agree with previous studies on the BPS exercise in the general population (Sheldon and Lyubomirsky, 2006; Peters et al., 2010), indicating that this exercise is also effective in inducing optimistic thinking and positive affect in patients with ED. Likewise, the absence of effects on negative affect found in this study is similar to results obtained in other trials with the BPS (Burton and King, 2004; Peters et al., 2010), suggesting that this exercise does not produce short-term effects on negative affect.

Given the potential benefits of the continued practice of the BPS exercise, the effects of the intervention over time were explored. Results showed that the BPS exercise produced larger decreases marginally significant in negative expectations after 1-month training compared to the control exercise. These effects are in line with prior results in general population (Meevissen et al., 2011; Enrique et al., 2017a), indicating that BPS exercise implemented through PT has the ability to decrease negative expectations in patients with ED. This is important given that these patients use to have a pessimistic view about the future (Malson et al., 2011), so that PPIs as the BPS exercise can produce benefits at this level. Contrary to our expectations, no statistical differences between conditions were found for the other primary and secondary outcomes when comparing the effects at post-training. Likewise, the analyses including the follow-up effects on the different outcomes did not show any statistically significant interaction between conditions. These results contradict some of the findings about the ability of BPS exercise to produce effects in future expectations, positive and negative affect, dispositional optimism and self-efficacy observed in general (Sheldon and Lyubomirsky, 2006; Meevissen et al., 2011) and clinical populations (Pietrowsky and Mikutta, 2012; Molinari et al., 2017).

Despite the absence of statistical differences between conditions, the average within-group effect size when combining the different measures was higher in the BPS condition than the control group across the different time frames. In this sense, the lack of statistically significant results could be explained by the fact that both conditions followed a trend toward improvement, explained by patients involvement in the psychological treatment for the ED, along with the small sample size, which might be complicating the emergence of significant results. Focusing on the difference between conditions in primary outcomes, future expectations and affect, both measures had a significant improvement over time. Indeed, future expectations was the variable that shed more pronounced differences between conditions, suggesting that BPS exercise was more effective on improving this measure, even reaching a large effect size on positive expectations subscale at the second follow-up. The cognitive nature of the BPS exercise (Erikson, 2007) could explain the larger effects in future expectations, in detriment of effects at an emotional level. In terms of affect, results were quite similar between conditions in terms of positive affect, suggesting that the change might be due to the treatment and not to a condition effect. Effects on negative affect were more pronounced in the BPS condition at long-term, suggesting that BPS exercise might had an influence in this decrease, although more studies are needed to confirm these trends. These results are not in line with prior literature which indicate that BPS exercise implemented with healthy and depressed samples have more impact on positive affect than on negative affect (Layous et al., 2012; Pietrowsky and Mikutta, 2012). However, the levels of negative affect in this population are higher than other clinical populations, which also allow a bigger room for improvement.

Looking at secondary measures, only general self-efficacy showed a significant improvement at post-training and results between conditions were quite similar, suggesting that the changes were due to the treatment and not to the condition. Dispositional optimism and hope showed non-significant changes in any of the conditions. These results could be explained by the fact that optimism and hope refer to personality traits that hardly can be changed (Pietrowsky and Mikutta, 2012). In this sense, even being non-significant, it is noticeable that results on these measures were slightly better in participants of the BPS condition, suggesting a positive trend that need to be confirmed by further studies with larger samples.

It is important to note that the BPS manipulation consisted of repeating the exercise of visualizing the best possible future over the training period and the follow-up based on the exercise developed in the first session (Enrique et al., 2017a). This situation could produce hedonic adaptation, meaning that the exercise no longer produces the same benefits observed at short-term (Diener et al., 2006). Different authors suggest that the integration of PPIs into more complex interventions would allow users to choose these strategies from a broader variety of exercises, avoiding the effects of the hedonic adaptation (Lyubomirsky and Layous, 2013). Therefore, it is possible to improve the efficacy of these interventions by combining different PPI and by introducing technologies as the ones displayed in this study (Enrique et al., 2017a; Molinari et al., 2017).

Although this study did not allow to draw conclusions about the role of the PT, its implementation was expected to make the exercise more rich and engaging. As a matter of fact, the patients included in this study were asked about their acceptability levels with the intervention and they informed adequate levels of satisfaction and usefulness. These results were published elsewhere (Enrique et al., 2017b). As Gaggioli et al. (2017) suggest, one of the goals of the PT is to improve the personal experience of the individuals by offering multisensorial experiences in which the content is offered through more than one senses, as the ones included in this trial. Future studies should explore if the inclusion of PT produces differential effects on the personal experience compared to the practice of the exercises without technologies.

This study has some limitations. First, the control condition focuses on the past as the participants had to think about the last 24 h, whereas the BPS exercise is future-oriented. Although other studies about BPS have used the same control condition (Sheldon and Lyubomirsky, 2006; Peters et al., 2010), future studies should include a control condition with the same temporal orientation in order to compare the results. Furthermore, given that it was established as a pilot study, sample size was not calculated and that lead to a little sample size (*N* = 54), which perhaps acted as a barrier for observing significant differences and affected to the generalization of the results. Still, our sample size was similar to other studies related to the field (Meevissen et al., 2011) and it was a clinically relevant sample. Regarding the technology used, it is important to note that the efficacy of the technologies was not compared to a condition without technologies, which means that we cannot know if technology is playing a role in the benefits obtained from the exercise. Another limitation is related to the description of the clinical sample because we did not collect information about the body mass index or the duration of the disorder, and both factors might influence the results obtained in this study. Yet, our results showed that severity of the ED pathology was not related to the change on the different outcome measures. Furthermore, half of the sample had a comorbid diagnosis of personality disorders, which keeps us from drawing conclusions only in terms of patients suffering from ED conditions. Thus, future studies should study the efficacy of PPIs in a sample with pure ED conditions in order to explore whether these PPIs act in the same way.

## Conclusion

This study illustrates the modest impact that a simple positive strategy implemented through PT has in patients with ED. This is the first study that tests the efficacy of the implementation of a PPI through PT in an ED sample. In this sense, it can serve as a reference for the design of new interventions aimed to improve well-being or quality of life on samples suffering ED. The trends observed in this study support the hypothesis that PPIs can act as supporting tools in the treatment of EDs by generating positive emotions that can protect against the harmful effects produced by ED conditions (Brannan and Petrie, 2011). However, more studies are needed to confirm these assumptions as there is a compelling need to provide these patients with positive resources in order to facilitate their recovery process (de Vos et al., 2017). Future studies should continue exploring the efficacy of different PPIs and their combination in ED samples in order to find out which strategies work better in what type of patients (Layous et al., 2012). If that should happen, it might contribute to generate additional resources in order to support the recovery process of patients with ED.

## Ethics Statement

This study was approved by the Research Ethics Committee of the Provincial Hospital of Castellón. All subjects gave written informed consent in accordance with the Declaration of Helsinki.

## Author Contributions

AE drafted the manuscript with important contributions from JB-L, FF-A, RB, and CB. AE in collaboration with GL, CB, and JB-L designed the study and participated in each of its phases. PR collaborated in the data analysis and the report of the results. GM collaborated in the manuscript development and participated in each study phase. VG and GL made important contributions in terms of sample recruitment. All authors participated in the review and revision of the manuscript and have approved the final manuscript to be published.

## Conflict of Interest Statement

The authors declare that the research was conducted in the absence of any commercial or financial relationships that could be construed as a potential conflict of interest.

## Footnotes

1 http://www.clinicaltrials.gov

2 https://www.teo.uji.es

## Abbreviations

ANOVA: analysis of variance
BPS: best possible self
DHS: Dispositional Hope Scale
EAT-26: Eating Attitudes Test
ED: eating disorder
GAF: Global Assessment of Functioning
GSES: General Self-Efficacy Scale
LOT-R: Life Orientation Test – Revised
PANAS: Positive and Negative Affect Scale
PPI: positive psychological intervention
PT: positive technology
SD: standard deviation
SPT: subjective probability task
